# Supplementary material for: Coactivators and general transcription factors have two distinct dynamic populations dependent on transcription
Source: EMBO J. 2017 Jul 19;36(18):2710–25. doi: 10.15252/embj.201696035 (PMC5599802; doi:10.15252/embj.201696035)
Supplement: Supplementary file 1 — Appendix [file EMBJ-36-2710-s001.pdf]

Appendix:

Coactivators and general transcription factors have two distinct dynamic populations dependent on transcription

Nikolaos Vosnakis[1,2,3,4,], **Marc Koch**[1,2,3,4], **Elisabeth Scheer**[1,2,3,4,], **Pascal Kessler**[1,2,3,4,], **Yves Mély**[4,7,], **Pascal Didier**[4,7,], **and László Tora**[1,2,3,4, *]

[1]Institut de Génétique et de Biologie Moléculaire et Cellulaire, Illkirch, France

[2]Centre National de la Recherche Scientifique, UMR7104, Illkirch, France

[3]Institut National de la Santé et de la Recherche Médicale, U964, Illkirch, France

[4]Université de Strasbourg, Illkirch, France

[7]Laboratoire de Biophotonique et Pharmacologie, Illkirch, France;

[*]Correspondence should be addressed to L.T. laszlo@igbmc.fr

Appendix Table of Content

Appendix Figure S1	pages 3-4
Appendix Figure S2	page 5
Appendix Figure S3	page 6
Appendix Figure S4	pages 7-8
Appendix Table S1	page 9
Appendix Supplementary Methods	pages 10-12
Appendix References	page 13

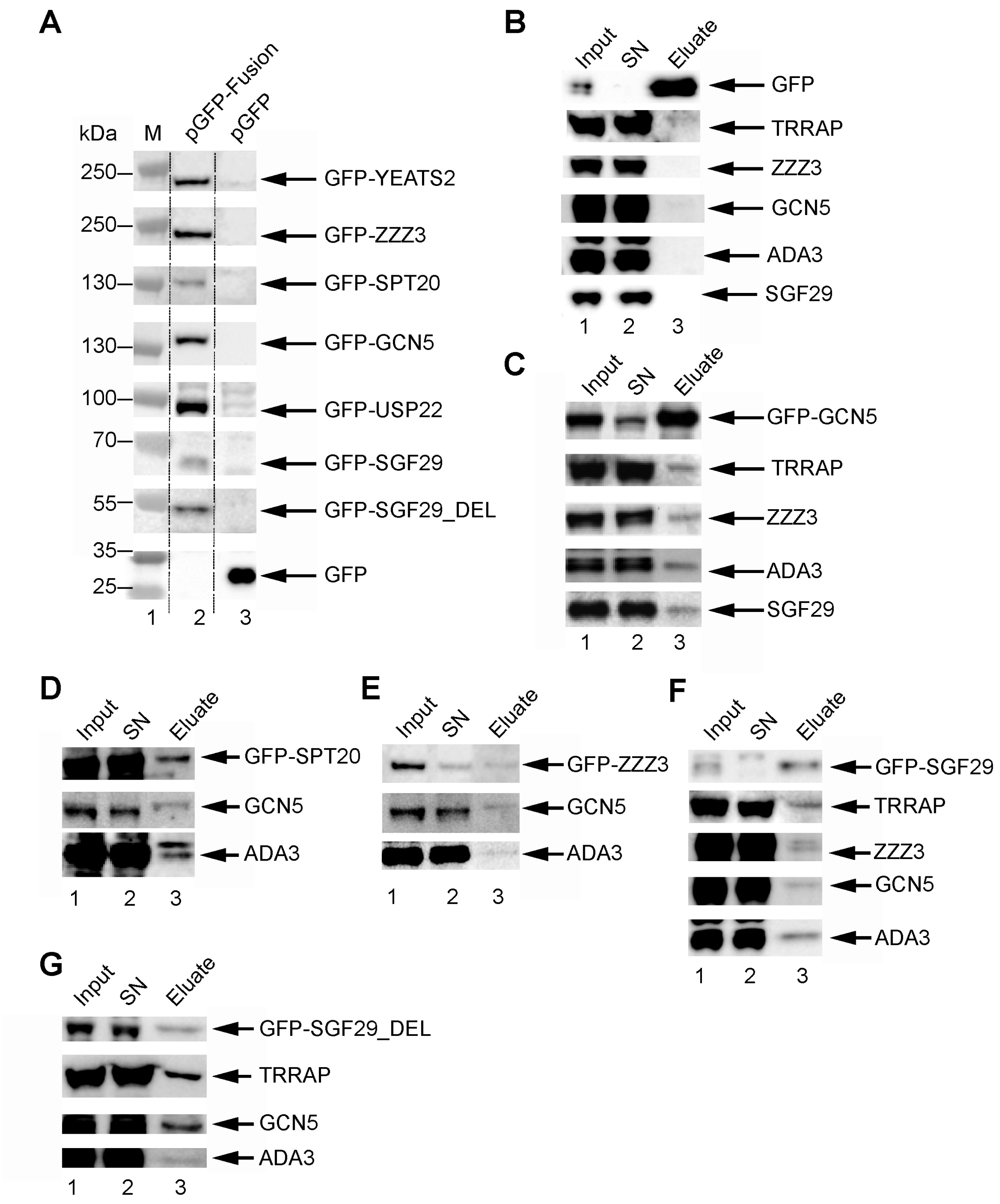

Appendix Figure S1. Biochemical characterisation of eGFP constructs

A) U2OS cells were transfected with the indicated eGFP fusion proteins (lane 2) or eGFP alone (lane 3) and whole cell extracts (WCEs) were prepared, 15 μg of WCE loaded in each case and GFP proteins size was analysed by Western blot (WB) using an anti-GFP antibody. Size marker (lane 1) indicates that each fusion protein was expressed at the expected size.

B-G) Anti-eGFP affinity purifications were performed on whole cell extracts of transfected cells using GFP-TrapA agarose beads. Co-immunoprecipitation of eGFP, eGFP-GCN5, eGFP-SPT20, eGFP-

ZZZ3 eGFP-SGF29, and SGF29-DEL with endogenous SAGA and/or ATAC subunits was analysed. Input (15 μg, 5%), Supernatant (SN; 15 μg, 5%) and eluate (25% of the IP) fractions were analysed with the indicated antibodies. **B**) Free eGFP does not interact with SAGA or ATAC subunits. The interactions of **C**) eGFP-GCN5, **D**) eGFP-SPT20, **E**) eGFP-ZZZ3, **F**) eGFP-SGF29 and **G**) eGFP-SGF29_DEL were analysed by western blot using the indicated antibodies.

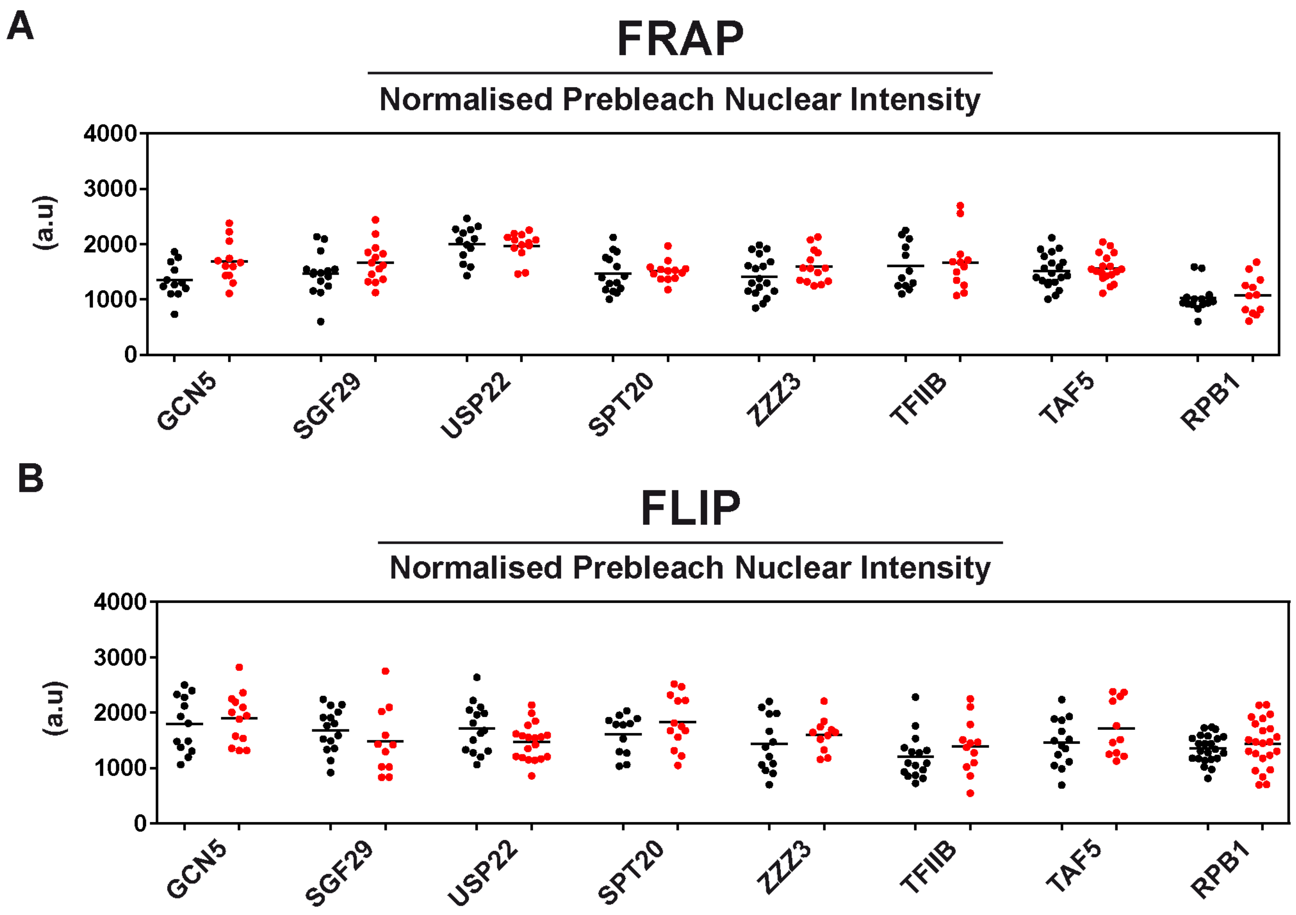

Appendix Figure S2. Cells selected for FRAP and FLIP measurements express the eGFP tagged factors at similar levels

Dot plots showing the average background-subtracted prebleached nuclear fluorescence intensity levels of individual cells selected for **A**) FRAP and **B**) FLIP measurements, in control conditions (black dots) or upon flavopiridol treatment (red dots). On the y axis fluorescence intensity is represented in arbitrary units (a.u). Horizontal bars represent mean values.

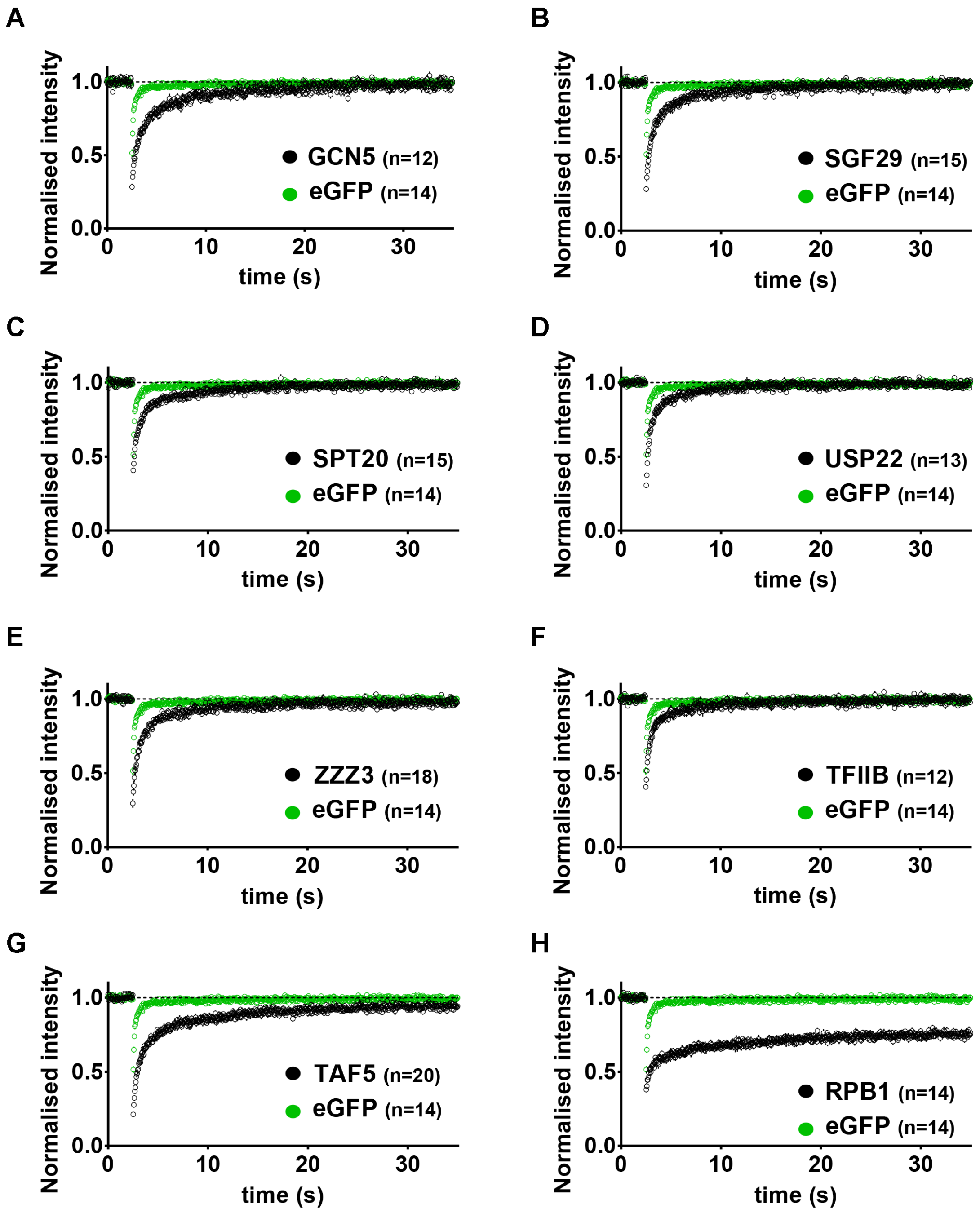

Appendix Figure S3. FRAP analyses suggest that SAGA and ATAC subunits and GTFs, TFIIB and TAF5, are highly mobile in live cell nuclei

Single normalised (Phair et al. 2004) FRAP curves. eGFP FRAP curve was compared to **A**) GCN5 and **B**) SGF29 (shared SAGA/ATAC subunits; **C**) SPT20 and **D**) USP22 (SAGA specific subunits); **E**) ZZZ3 (ATAC specific subunit), **F**) TFIIB, **G**) TAF5 (TFIID subunit) and **H**) RPB1 (Pol II subunit).

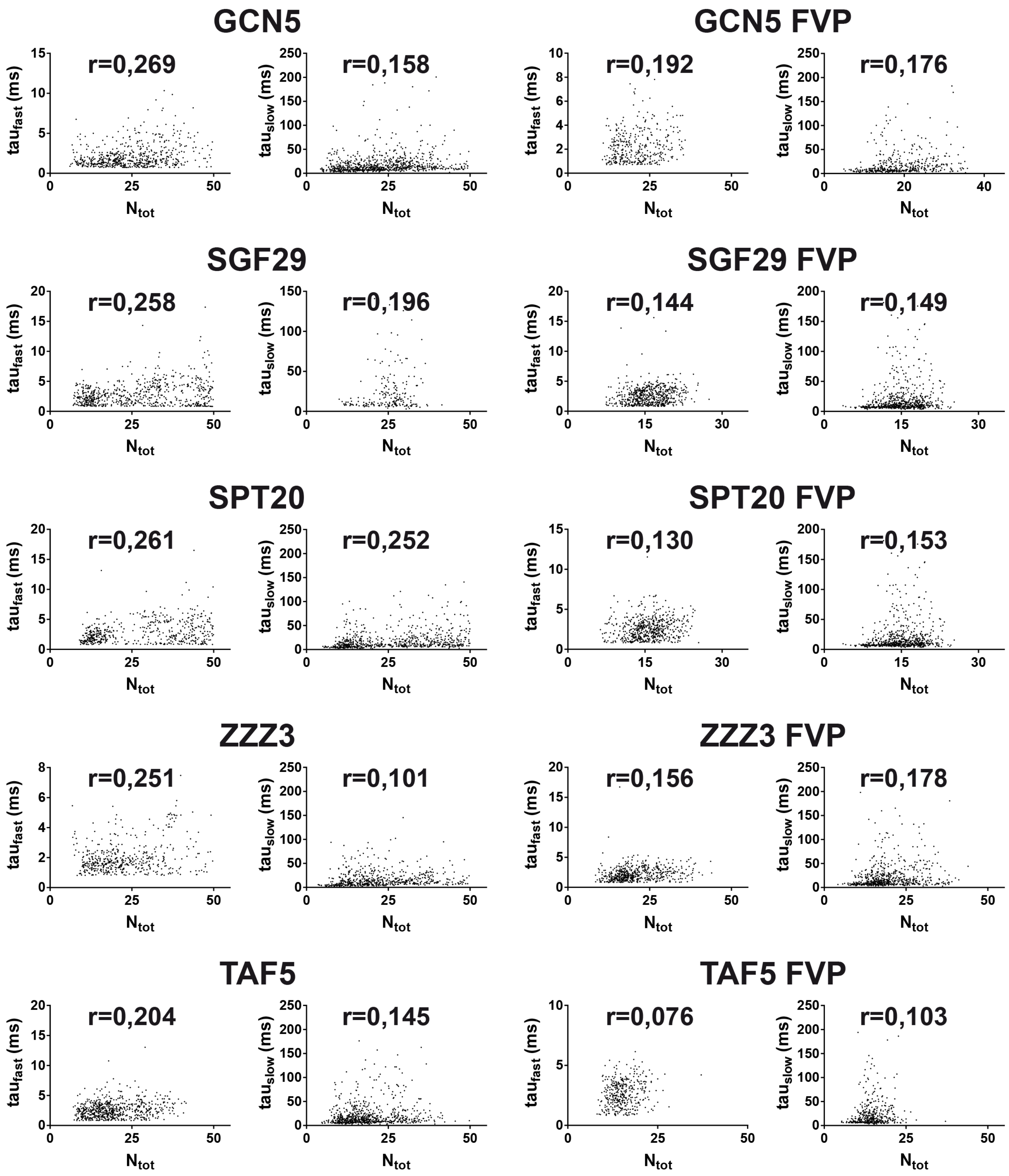
GCN5
r=0,269
r=0,158
GCN5 FVP
r=0,192
r=0,176
SGF29
r=0,258
r=0,196
SGF29 FVP
r=0,144
r=0,149
SPT20
r=0,261
r=0,252
SPT20 FVP
r=0,130
r=0,153
ZZZ3
r=0,251
r=0,101
ZZZ3 FVP
r=0,156
r=0,178
TAF5
r=0,204
r=0,145
TAF5 FVP
r=0,076
r=0,103
tau_{fast} (ms)
tau_{slow} (ms)
N_{tot}

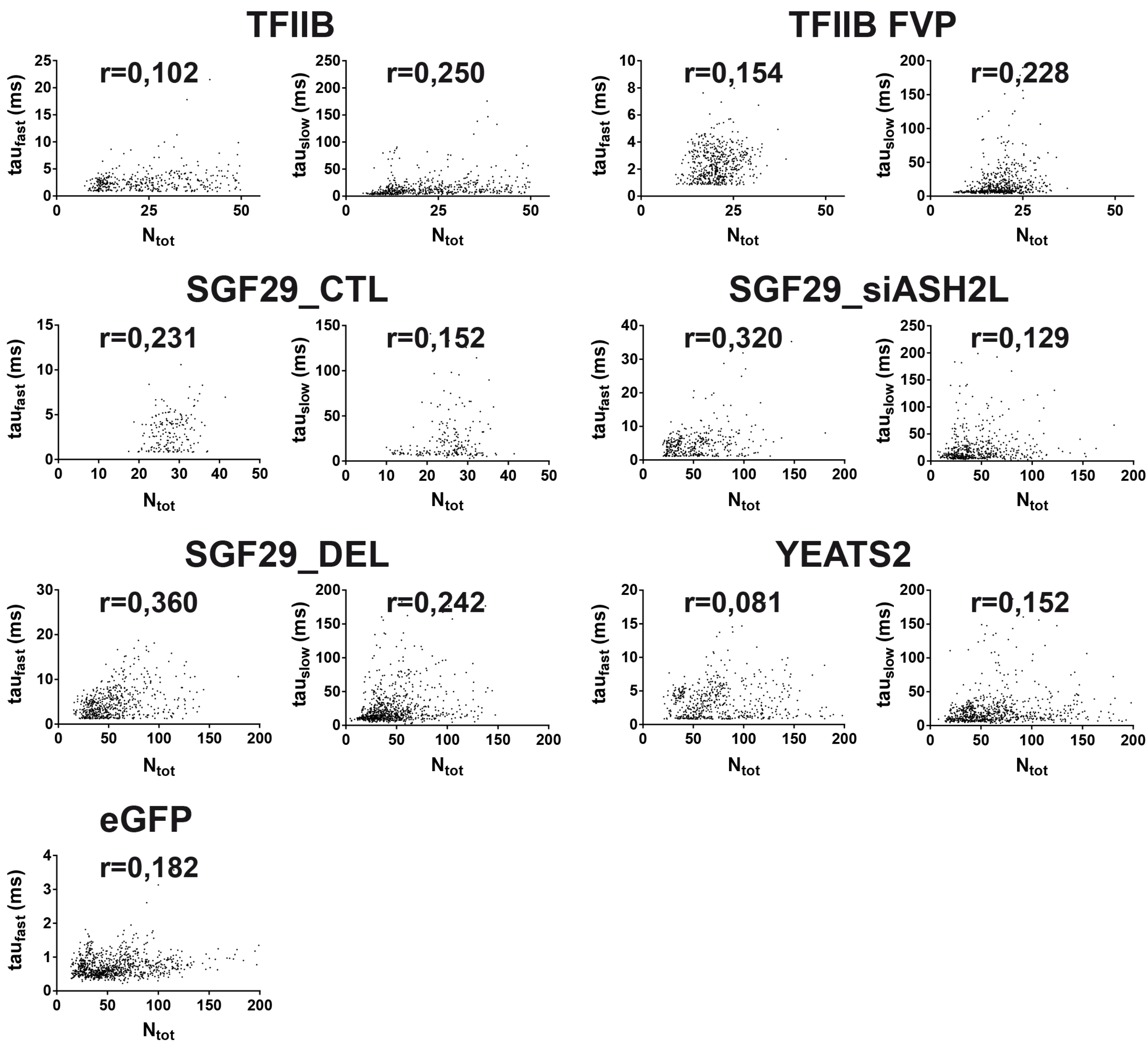

Appendix Figure S4. The selected expression level of eGFP tagged factors does not affect the estimation of diffusion parameters by FCS

The estimated diffusion time of the fast (tau_{fast}) or slow (tau_{slow}) component of eGFP tagged factors, in control conditions or upon flavopiridol treatment, is plotted versus the total number of molecules (N_{tot}) present in the observation volume. Pearson correlation coefficient (r) between each pair of variables is indicated on each graph. In all cases, very low or no correlation was found between N_{tot} and tau_{fast} or tau_{slow}.

	Normal	+Flavopiridol
**GCN5**	9.80E-07	5.50E-07
**SGF29**	2.90E-08	7.60E-09
**SGF29_DEL**	7.10E-08	-
**SPT20**	4.80E-08	1.49E-07
**ZZZ3**	2.60E-08	6.60E-08
**YEATS2**	6.20E-08	6.80E-08
**TAF5**	6.70E-08	9.00E-08
**TFIIB**	5.40E-08	3.70E-08

Appendix Table S1

Reduced chi square values obtained from the two populations fit corresponding to all the studied factors under normal conditions (see Figure 5) or in the presence of Flavopiridol (see Figure 6). The values obtained from the one population fit are an order of magnitude higher while the three populations fit provides only a gain by a factor of two. Together with the maximum entropy algorithm these values further support the two populations model.

Appendix Supplementary Methods

Cell culture

U2OS, human osteosarcoma cell line was grown at 37˚C in 5% CO2 in Dulbecco's Modified Eagle's medium (DMEM) (Invitrogen) supplemented with 1 g/l glucose, 10% foetal calf serum (FCS) and gentamycin (40 µg/ml). HEK293, human embryonic kidney cells were grown in DMEM (1g/l glucose), 10% FCS and penicillin (100 Units/ml) streptomycin (100 µg/ml) at 37°C, in 5% CO2.

eGFP constructs

cDNAs encoding hGCN5 (Demeny et al. 2007), hSPT20 (Nagy et al. 2009), hZZZ3 (obtained from OriGene Technologies, Inc), hSGF29, hYEATS2 (kind gift from Z. Nagy and A. Riss), were PCR amplified and N-terminally fused to eGFP coding sequence, previously inserted in pcDNA™3.1(+) (Invitrogen) vector. For the eGFP-NLS construct, a 3 x NLS coding sequence was added at the C-terminus of eGFP by PCR and subcloned in pcDNA™3.1(+). TFIIB encoding cDNA (Moncollin et al. 1992) was PCR amplified and C-terminally fused with sequences coding for eGFP, previously inserted in pcDNA™3.1(+). The eGFP-USP22 construct in pcDNA3.1 was described in (Umlauf et al. 2013). pBABE vector expressing eGFP-TAF5, described in (de Graaf et al. 2010) was used as PCR template to amplify the cDNA encoding eGFP-TAF5 which was inserted in pcDNA3.1. The construct expressing eGFP-RPB1 has been described in (Sugaya et al. 2000). To generate the eGFP-SGF29_DEL truncated mutant (lacking the tandem tudor domains of SGF29), a PCR amplified fragment corresponding to the first 159 amino acids of hSGF29 cDNA was N-terminally fused to eGFP coding sequences in pcDNA3.1. All constructs were verified by DNA sequencing, performed at GATC Biotech (Germany) and the expression of the fusion proteins at the expected size was tested by Western blot.

Plasmid DNA and siRNA transfection

For plasmid DNA (pcDNA3.1 coding the indicted factors) transfections for FRAP, FLIP and FCS experiments, 1,5-2 ×105 cells were seeded on 35 mm diameter, high Glass Bottom µ-Dishes (Ibidi, #81158) and transfected before they were completely attached using FuGENE 6 transfection reagent (Promega, #E269A) according to manufacturer's instructions. Since the transfected amounts of the eGFP constructs were low, between 25 and 50 ng, depending on the fusion protein to be expressed, plasmid DNA amounts were in all cases supplemented with empty pcDNA™3.1(+) (backbone vector) to a final amount of 1.5 µg/35 mm diameter plate. Transfected amounts of eGFP tagged factors were different for each technique: FRAP/FLIP: 10-50 ng/35 mm plate. FCS: 5-20 ng/35 mm plate. Cells were imaged 16-24 hours after transfection.

For siRNA mediated knockdown experiments, 150-200 pmol of siASH2L (Dharmacon, #L-019831-00) or non-targeting siControl (Dharmacon, #D-001206-13) were used to transfect U2OS cells using Lipofectamine® 2000 (Invitrogen, # 116680). Cell were transfected directly after seeding in 35 mm petri dishes and knockdown efficiency was evaluated 72 h after transfection. For imaging experiments, cells were initially transfected with the respective siRNA in 35 mm dishes, trypsinized after 48 h and seeded in Ibidi μ (35mm) high glass bottom dishes. Cells were then transfected with pcDNA3.1 plasmids coding for the indicated factors and imaging experiments were conducted 16-24 h later (total of 72h upon siRNA transfection).

Preparation of protein extracts

Acidic extracts were performed as described in (Lang et al. 2011). Briefly, cell pellets were resuspended in 5 volumes (V) of acidic extraction buffer: 10 mM HEPES (pH 7.9), 1.5 mM MgCl2, 10 mM KCl, 0.5 mM dithiothreitol (DTT), N-Ethylmaleimide (NEM) (Sigma-Aldrich, #E1271) supplemented with Protease Inhibitor Cocktail (PIC; Roche, #11873580001) and HCl to a final concentration of 0.2 M. After 30 minutes incubation, the extracts were centrifuged at 12,000 g (10 min, 4oC). Supernatants were transferred to a clean tubes. Prior SDS-PAGE analysis, 1/10 volume of Tris-HCl 2M (pH 8.8) was added to each sample.

Whole cell extracts (WCEs) were prepared as previously described (Zhao et al. 2008). In short, cell pellets were suspended in protein extraction buffer 20 mM Tris-HCl (pH 7.5), 2 mM DTT, 20 % glycerol, 400 mM KCl and PIC. Samples were rapidly frozen in liquid nitrogen and slowly thawed on ice, and the freeze/thawing cycles repeated 3 times. Supernatants containing the protein extract were collected following centrifugation at 12,000 g (15 min, 4 °C).

Subcellular fractionation was performed as described in (Bleuyard et al. 2012) with minor modifications. 0.5-1.0 × 107 cells were washed and scrapped with ice-cold PBS supplemented with PIC. Cells were centrifuged at 200 g 3 min 4°C and the pellets were resuspended in 5 volumes sucrose buffer (10 mM Tris pH 7.5, 20 mM KCl, 250 mM sucrose, 2.5 mM MgCl2, and PIC).Triton X-100 was added to a final concentration of 0.2 % and cells were subsequently vortexed three times for a total of 30 seconds. Suspensions were centrifuged at 500 g (5 min, 4°C) to collect the supernatant, corresponding to the cytoplasmic fraction. Nuclei were incubated on ice for 30 min in 5 volumes of NETN150 buffer (50 mM Tris pH 8.0, 150 mM NaCl, 2 mM EDTA, 0.5% NP-40 and PIC). The nuclear soluble fraction was collected upon centrifugation at 1000 g (5 min, 4ºC). The chromatin-enriched pellet was incubated on ice for ~1,5 h with 125 U/ml BaseMuncher (Expedeon, BM0025) in NETN150 buffer supplemented with 2 mM MgCl2. Chromatin associated fraction was collected upon centrifugation at 16000 g (30 min, 4°C). Soluble nuclear and chromatin associated fractions were concentrated using 3K Amicon® Ultra centrifugal filters centrifuged at 14000 g (15-30 min, 4°C).

Western blot assays

Western blot assays were performed according to standard protocols. Briefly, protein samples were separated by 10% SDS-PAGE (or 6% for the efficient separation of Pol IIO and Pol IIA forms), and transferred to nitrocellulose membranes (Whatman). Membranes were blocked with 3% skimmed milk in 1xPBS for 1 hour at room temperature. Incubation of the membranes with the different primary antibodies diluted in 0.3% skimmed milk in 1xPBS was usually performed over night at 4°C. Membranes were washed (3 x 10 min) with 0.05% Tween in 1xPBS and incubated (1 h) with anti-mouse (GAMpo) or rabbit (GARpo) horseradish peroxidase linked secondary antibody (1:10000 dilution) from Jackson Laboratories. Enhanced chemiluminescence (ECL) detection system (GE Healthcare) was used to reveal antigen-antibody complexes.

Immunoprecipitations

For eGFP affinity purifications, U2OS cells were transfected in 10 cm diameter dishes using 50-100 ng of plasmid DNA per dish. 0.5 mg of whole cell extracts were used to immunoprecipitate eGFP alone or eGFP-tagged proteins using GFP-Trap®_A agarose beads (Chromotek, GTA) according to manufacturer's protocol with minor modifications. Briefly, input samples were diluted to a final concentration of 100 mM KCl in IP0 buffer (25 mM Tris-HCl pH 7.9, 0.1% NP40, 5 mM MgCl2, 10 % glycerol, 2 mM DTT, supplemented with PIC). GFP-Trap beads suspension were washed 3 times in Milli-Q H2O. Input samples were incubated with 1/20 of input volume of beads for 1-1.5 h at 4°C. Beads were washed 3 × 5 min with IP150 buffer (IP0 buffer containing 150 mM KCl). Immunoprecipitated proteins were eluted from the beads with 2 volumes of 0.1 M glycine (pH 2.3) at 4°C for 1 min. Eluates were neutralized with 1/10 volume of 2 M Tris-HCl (pH 8.8).

Appendix References

Bleuyard, JY, Buisson, R, Masson, JY & Esashi, F (2012) ChAM, a novel motif that mediates PALB2 intrinsic chromatin binding and facilitates DNA repair. *EMBO reports* 13:135-41. DOI: 10.1038/embor.2011.243.

de Graaf, P, Mousson, F, Geverts, B, Scheer, E, Tora, L, Houtsmuller, AB & Timmers, HT (2010) Chromatin interaction of TATA-binding protein is dynamically regulated in human cells. *Journal of cell science* 123:2663-71. DOI: 10.1242/jcs.064097.

Demeny, MA, Soutoglou, E, Nagy, Z, Scheer, E, Janoshazi, A, Richardot, M, Argentini, M, Kessler, P & Tora, L (2007) Identification of a Small TAF Complex and Its Role in the Assembly of TAF-Containing Complexes. *PLoS ONE* 2:e316.

Lang, G, Bonnet, J, Umlauf, D, Karmodiya, K, Koffler, J, Stierle, M, Devys, D & Tora, L (2011) The tightly controlled deubiquitination activity of the human SAGA complex differentially modifies distinct gene regulatory elements. *Molecular and cellular biology* 31:3734-44. DOI: 10.1128/MCB.05231-11.

Moncollin, V, Fischer, L, Cavallini, B, Egly, JM & Chambon, P (1992) Class II (B) general transcription factor (TFIIB) that binds to the template-committed preinitiation complex is different from general transcription factor BTF3. *Proc.Natl.Acad.Sci.U.S.A.* 89:397-401.

Nagy, Z, Riss, A, Romier, C, le Guezennec, X, Dongre, AR, Orpinell, M, Han, J, Stunnenberg, H & Tora, L (2009) The human SPT20-containing SAGA complex plays a direct role in the regulation of endoplasmic reticulum stress-induced genes. *Mol Cell Biol* 29:1649-60.

Phair, RD, Gorski, SA & Misteli, T (2004) Measurement of dynamic protein binding to chromatin in vivo, using photobleaching microscopy. *Methods Enzymol* 375:393-414.

Sugaya, K, Vigneron, M & Cook, PR (2000) Mammalian cell lines expressing functional RNA polymerase II tagged with the green fluorescent protein. *J Cell Sci* 113:2679-83.

Umlauf, D, Bonnet, J, Waharte, F, Fournier, M, Stierle, M, Fischer, B, Brino, L, Devys, D & Tora, L (2013) The human TREX-2 complex is stably associated with the nuclear pore basket. *Journal of cell science* 126:2656-67. DOI: 10.1242/jcs.118000.

Zhao, Y, Lang, G, Ito, S, Bonnet, J, Metzger, E, Sawatsubashi, S, Suzuki, E, Le Guezennec, X, Stunnenberg, HG, Krasnov, A, Georgieva, SG, Schule, R, Takeyama, K, Kato, S, Tora, L & Devys, D (2008) A TFTC/STAGA module mediates histone H2A and H2B deubiquitination, coactivates nuclear receptors, and counteracts heterochromatin silencing. *Mol Cell* 29:92-101.
