# Supplementary material for: Coactivators and general transcription factors have two distinct dynamic populations dependent on transcription
Source: EMBO J. 2017 Jul 19;36(18):2710–25. doi: 10.15252/embj.201696035 (PMC5599802; doi:10.15252/embj.201696035)
Supplement: Supplementary file 2 — Expanded View Figures PDF [file EMBJ-36-2710-s002.pdf]

Expanded View Figures

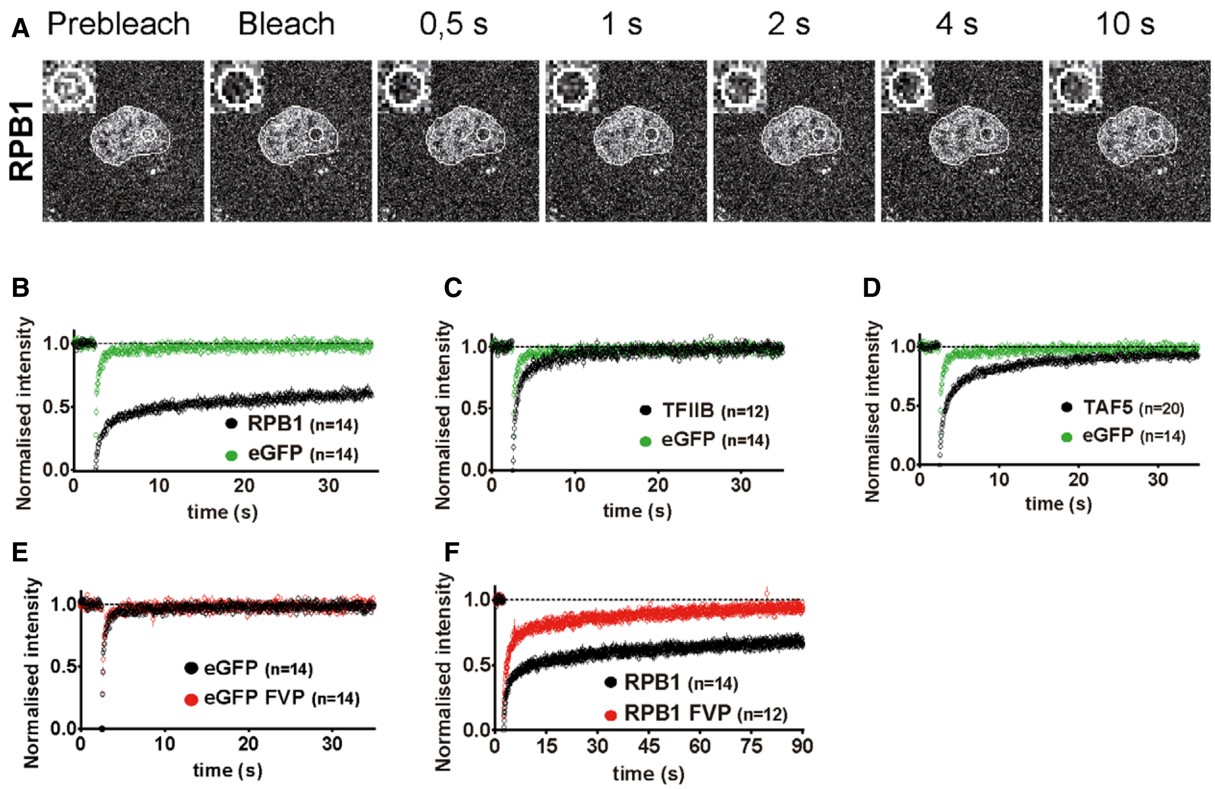

Figure EV1. FRAP analyses suggest that SAGA and ATAC subunits and GTFs, TFIIB and TAF5, are highly mobile in live-cell nuclei.

A FRAP experiments performed on eGFP-RPB1-expressing human U2OS cells. Representative images of the photobleached nuclear regions are shown. Time after photobleaching is indicated on top of the panels. An inset, showing a four times zoom-in of the bleached ROI (white circle) is shown at the top left corner of each frame. The nucleus of each cell is encircled with a white line. Scale bar is 20 μm, as shown in Figure 1A.

B–D Average normalized FRAP curve of eGFP was compared to (B) RPB1 (Pol II subunit); (C) TAF5 (TFIID subunit); (D) TFIIB.

E, F Comparison of FRAP curves obtained from control and flavopiridol (FVP)-treated cells corresponding to (E) eGFP; and (F) RPB1 are also shown.

Data information: On the *x*-axis, time is represented in seconds (s). *n*: number of cell nuclei analysed for each factor.

Figure EV2. FLIP analyses indicate transient chromatin interaction of SAGA and ATAC and PIC components, TAF5 and TFIIB, over the whole nucleus.

A FLIP experiments performed on eGFP-NLS-, eGFP-GCN5- and eGFP-RPB1-expressing cells. Representative images of cell nuclei subjected to FLIP are shown. The respective time points of FLIP experiments are indicated on top of the panels. The repeatedly bleached nuclear ROI (white circle) and the whole nuclei (encircled with a white line) of the cells are marked. The scale bar is 20 μm.

B–I Average normalized FLIP curves of eGFP-NLS were compared to (B) RPB1 (Pol II subunit); (C) TAF5 (TFIID subunit); (D) TFIIB; (E) GCN5 and (F) SGF29 (shared SAGA and ATAC subunits); (G) SPT20 and (H) USP22 (SAGA subunits); (I) ZZZ3 (ATAC subunit).

J–M Overlay of FLIP curves of (J) SPT20 (SAGA) and ZZZ3 (ATAC); (K) GCN5 (shared SAGA and ATAC subunit) and TAF5 (TFIID subunit); (L) RPB1 (Pol II subunit) and GCN5 (shared SAGA and ATAC subunit); and (M) RPB1 (Pol II subunit) and TAF5 (TFIID subunit).

Data information: Graphs represent average normalized values of nuclear fluorescence intensities in the whole nucleus over time. Error bars show ± SD from the mean. On the *x*-axis, time is represented in minutes (min). *n*: number of cells analysed for each factor.

© 2017 The Authors

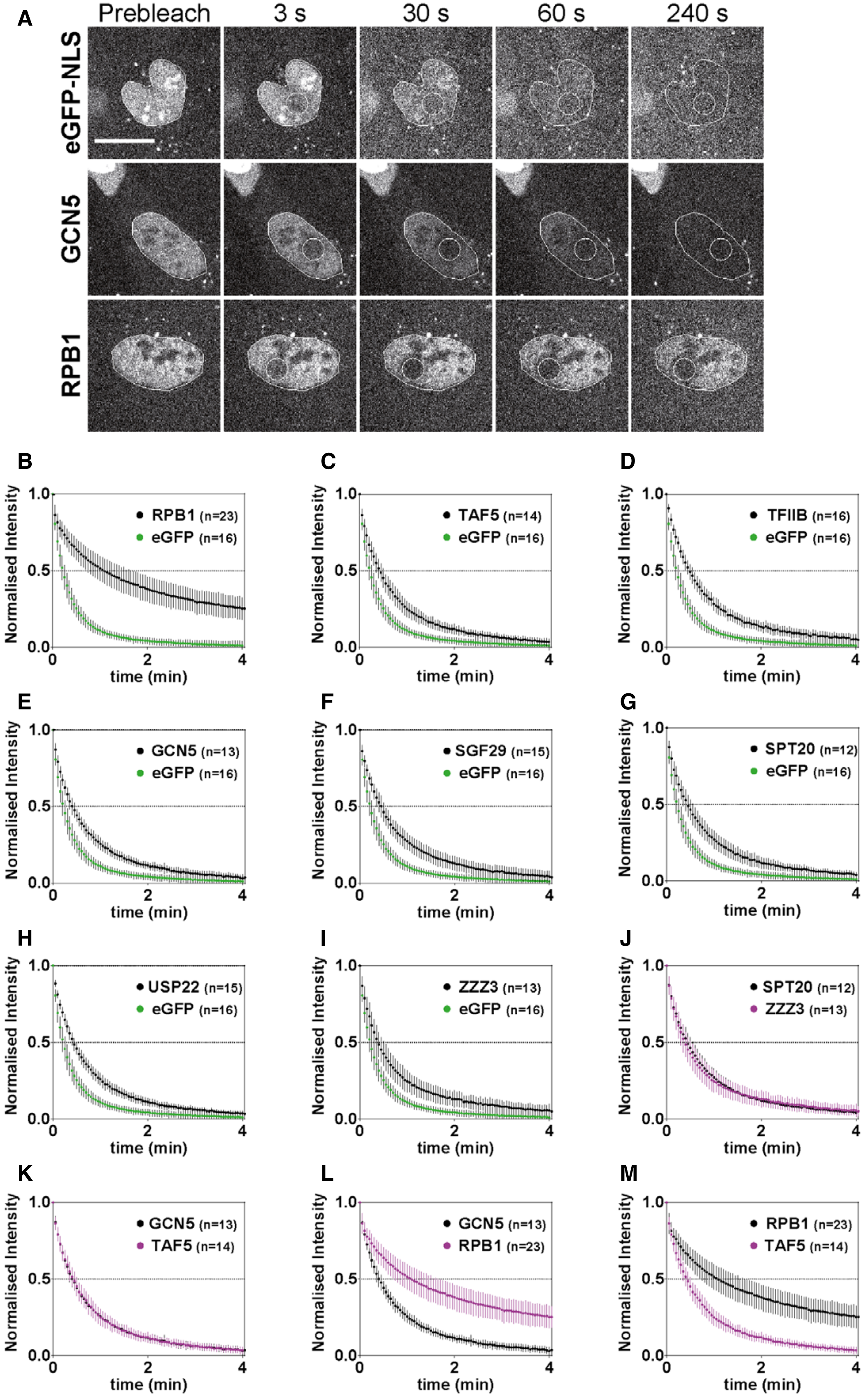

Figure EV2.

 © 2017 The Authors

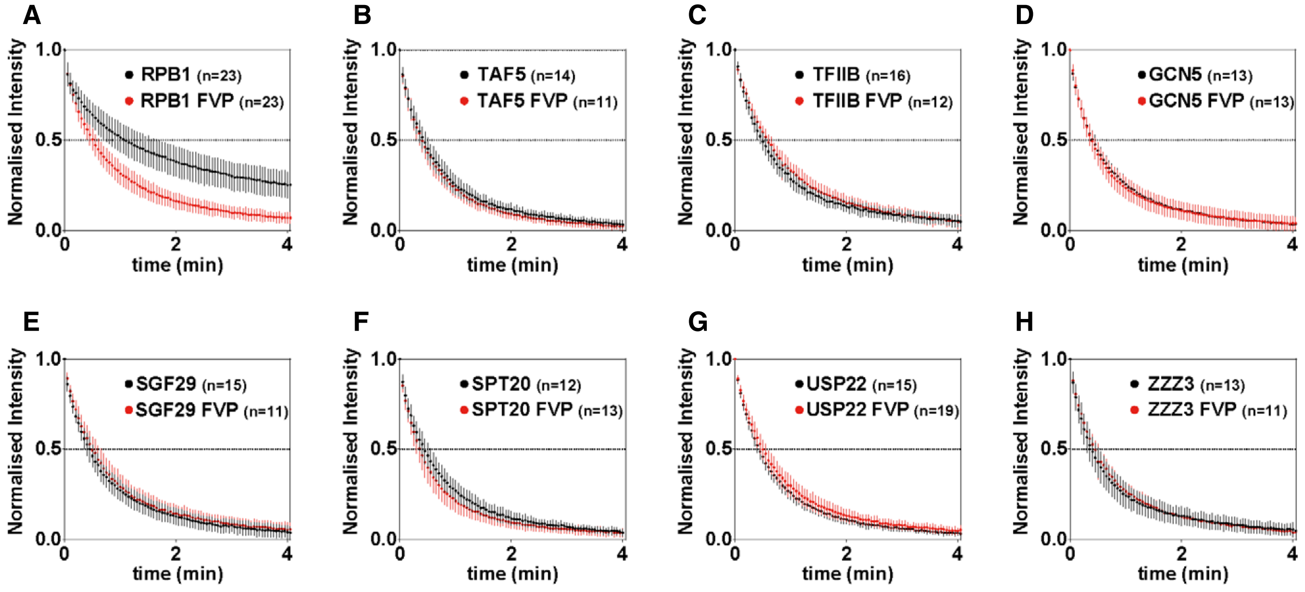

Figure EV3. Nuclear FLIP analysis in control and flavopiridol-treated cell shows no significant change in the dynamics of TAF5, TFIIB, SAGA and ATAC subunits over the whole nucleus.

A–H Average normalized FLIP curves from measurements in control and FVP-treated cells corresponding to the following factors: (A) RPB1 (Pol II subunit); (B) TAF5; (C) TFIIB and (D) GCN5 and (E) SGF29 (shared SAGA and ATAC subunits); (F) SPT20 and (G) USP22 (SAGA subunits); (H) ZZZ3 (ATAC subunit). All graphs represent the average normalized values of nuclear fluorescence intensity in the whole nucleus over time. Error bars show ± SD from the mean. Black points: average FLIP curves derived from measurements in control cells. Red points: average FLIP curves in flavopiridol-treated cells. On the *x*-axis, time is represented in minutes (min). *n*: number of cells analysed for each factor.

© 2017 The Authors
