# Supplementary figures and images for: Coactivators and general transcription factors have two distinct dynamic populations dependent on transcription
Source: EMBO J. 2017 Jul 19;36(18):2710–25. doi: 10.15252/embj.201696035 (PMC5599802; doi:10.15252/embj.201696035)

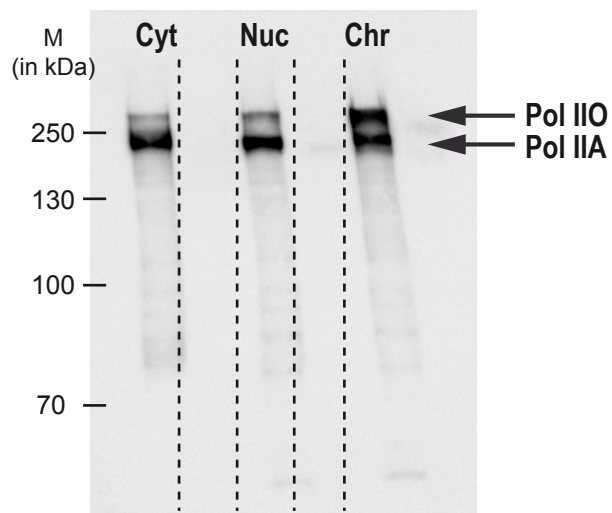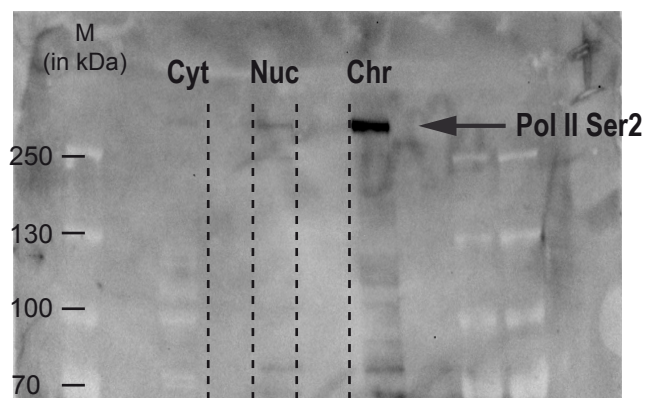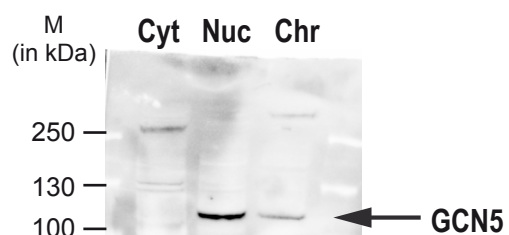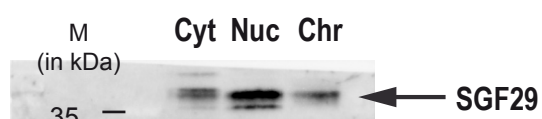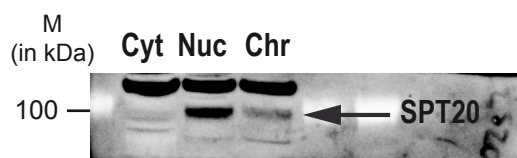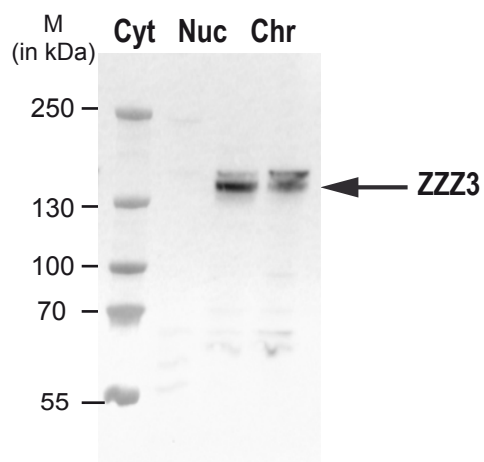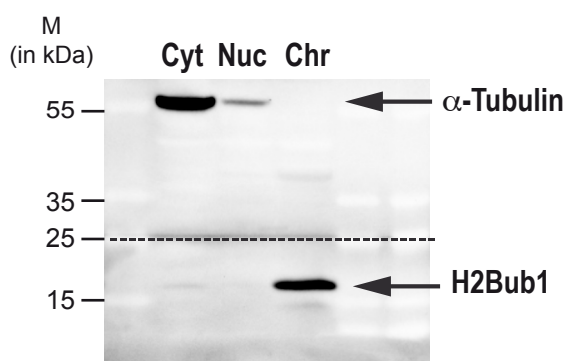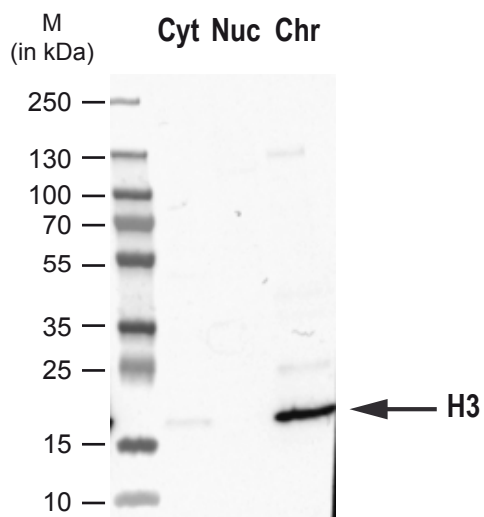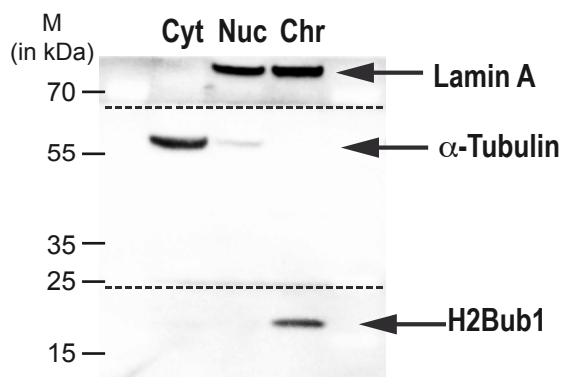

Supplement: Supplementary file 4 — Source Data for Figure 2 [file EMBJ-36-2710-s003.pdf]

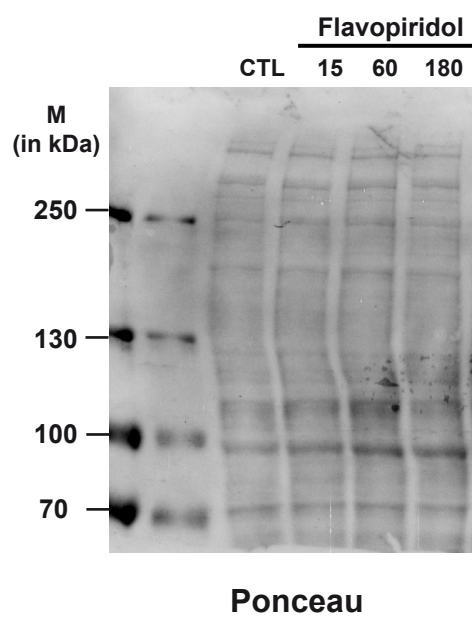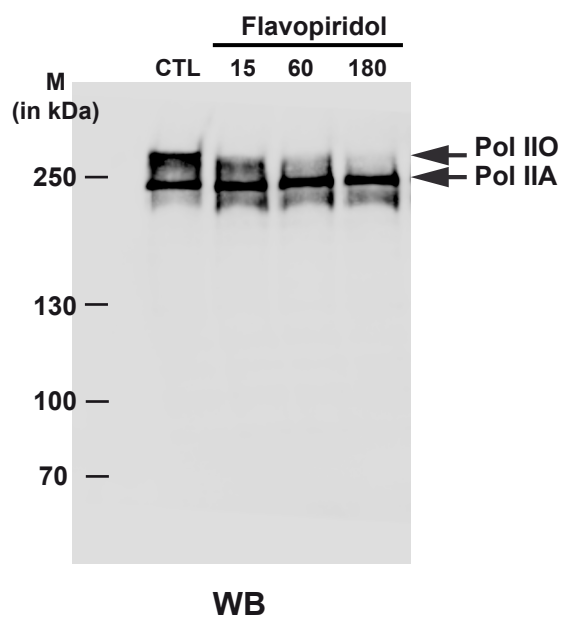

Supplement: Supplementary file 5 — Source Data for Figure 3 [file EMBJ-36-2710-s004.pdf]

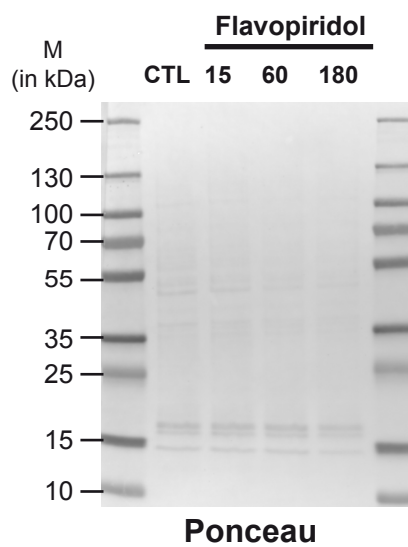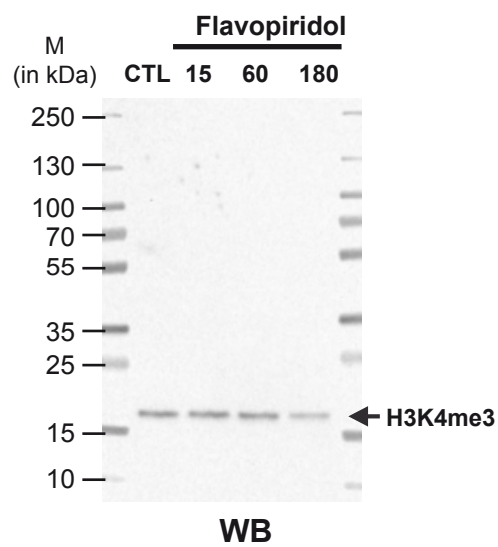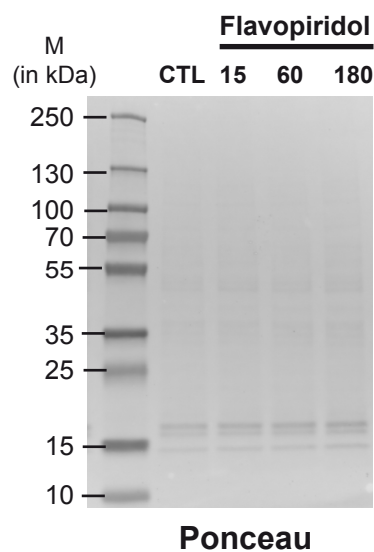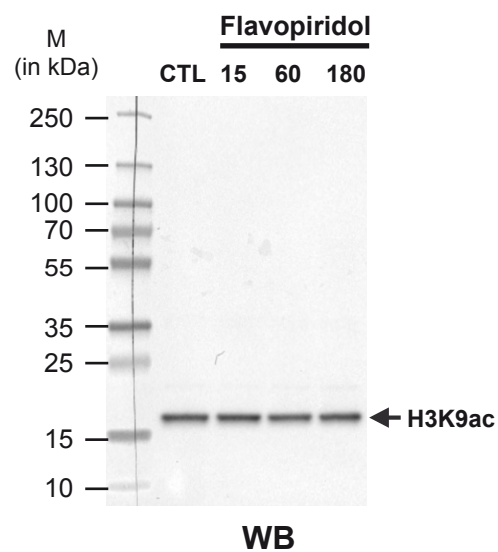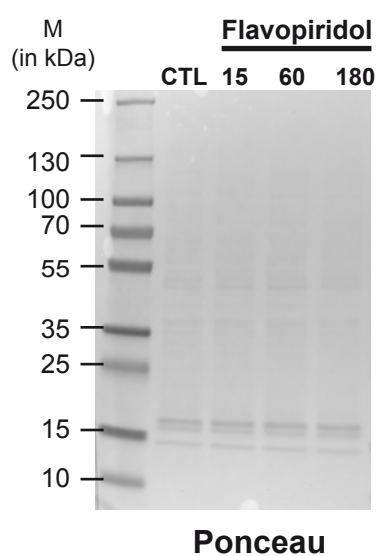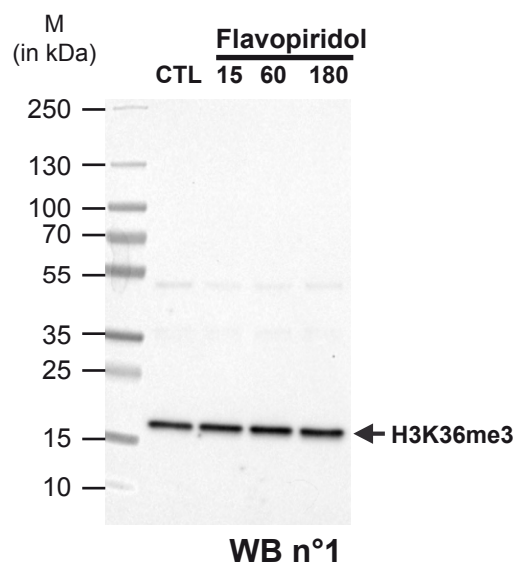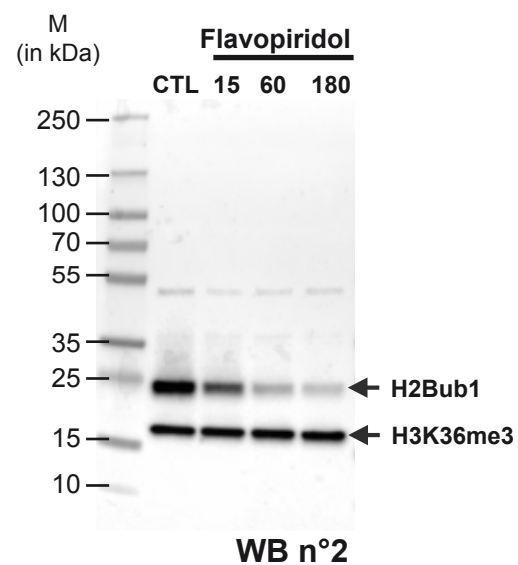

Supplement: Supplementary file 6 — Source Data for Figure 7 [file EMBJ-36-2710-s005.zip › 96035_Sourcedata_7A.pdf]

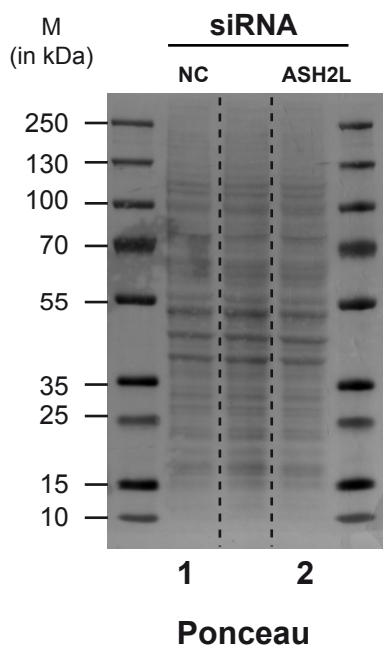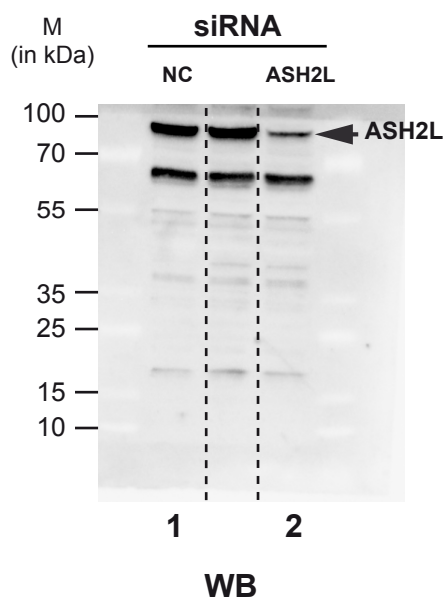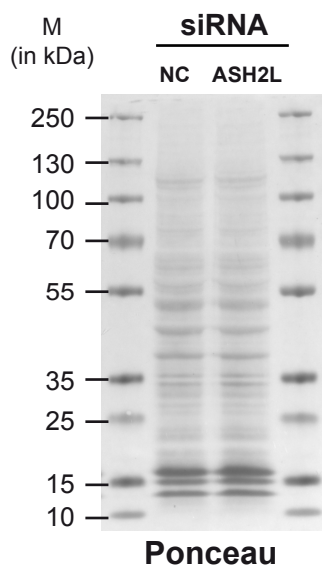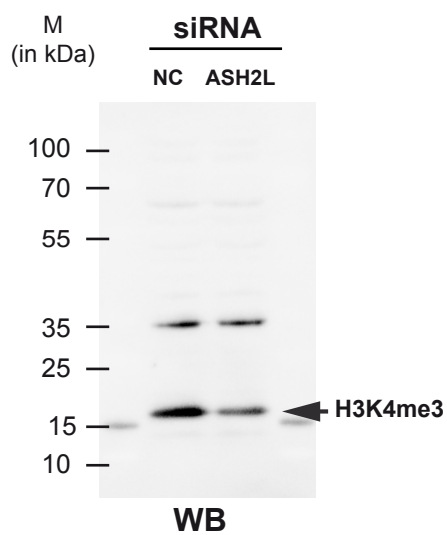

Supplement: Supplementary file 6 — Source Data for Figure 7 [file EMBJ-36-2710-s005.zip › 96035_Sourcedata_7C.pdf]
